# Prevalence and risk factors of self-reported wrist and hand symptoms and clinically confirmed carpal tunnel syndrome among office workers in China: a cross-sectional study

**DOI:** 10.1186/s12889-020-10137-1

**Published:** 2021-01-06

**Authors:** Beibei Feng, Kedi Chen, Xiaoxia Zhu, Wing-Yuk Ip, Lars L. Andersen, Phil Page, Yuling Wang

**Affiliations:** 1 Department of Rehabilitation Medicine, The Sixth Affiliated Hospital of Sun Yat-sen University, Guangzhou, 510655 China; 2 Department of Orthopaedics & Traumatology, The University of Hong Kong, Hong Kong, SAR China; 3 National Research Centre for the Working Environment, Copenhagen, Denmark; 4 Doctor of Physical Therapy Program, Franciscan Missionaries of Our Lady University, Baton Rouge, Louisiana, USA

**Keywords:** Median nerve, Office workplace, Computer use, Work in pain, Work-related musculoskeletal disorder, Wrist and hand complaint

## Abstract

**Background:**

Carpal tunnel syndrome (CTS) is a common cause of pain, numbness and tingling in the wrist and hand region and is associated with repetitive wrist and hand use in office workers. However, scarce knowledge exists about the epidemiology of clinically confirmed CTS among Chinese office workers. This study aimed to investigate the prevalence of wrist/hand symptoms and CTS in office workers in China and to identify associated risk factors.

**Methods:**

A cross-sectional survey was carried out in a metropolitan city in China involving 969 respondents (aged 17–49 years) from 30 workplaces. A questionnaire was distributed to each participant to collect their demographic, work-related physical and psychosocial factors, and wrist and hand symptoms. The wrist and hand pain/numbness symptoms were marked on a body chart and the nature and intensity of symptoms, nocturnal symptoms, as well as aggravating activities were also recorded. Clinically confirmed CTS cases were screened based on the history, Phalen’s test, Tinel Sign and skin sensation testing among symptomatic respondents. Logistic regression was employed to estimate the odds ratio (OR) and 95% confidence interval (95% CI) for the occurrence of self-reported wrist and hand symptoms and clinically confirmed CTS.

**Results:**

The clinically confirmed CTS prevalence was 9.6%. The prevalence of wrist and hand symptoms were 22 and 15%, respectively. Frequently working in pain was associated with higher odds of CTS. Multivariate modelling adjusted for age and gender showed that prolonged computer use time and working without breaks were associated with presence of wrist/hand symptoms (adjusted ORs: 1.11 (95% CI 1.02–1.22) and 1.88 (95% CI 1.12–3.14)). Educational level was inversely associated with CTS and smoking was associated with wrist/hand complaints (adjusted OR: 2.20 (95% CI 1.19–4.07)).

**Conclusions:**

The prevalence of work-related clinically confirmed CTS symptoms among young office workers in China is high. Frequently working in pain is closely associated with clinically confirmed CTS. Intense computer use and no breaks at work are associated with wrist and hand symptoms.

## Background

Carpal tunnel syndrome (CTS), caused by compression of the median nerve within the carpal tunnel, is the most prevalent peripheral nerve entrapment disease, affecting thousands of citizens worldwide [1–4]. The prevalence of CTS has been reported to be around 5% in the general population [1, 4–6]. The risk factors for CTS include repeated and excessive use of the wrist and hand, awkward postures, heavy lifting, and vibrations as well as personal predictors as female gender, obesity, older age, and smoking [3, 4, 7–11]. Different occupational populations involving the above risk factors may be susceptible to CTS development. Work-related CTS prevalence in different occupational studies varies, ranging from 1 to 61% [2, 4, 6, 12]. The highest CTS prevalence of 61% was found among industrial workers mainly using grinding tools, while about 1% of industrial workers with forceful but low repetitive hand use developed CTS [12]. The disabling symptoms associated with CTS result in surprisingly high expenditures in both medical and non-medical costs [3, 13, 14]. CTS has been perceived as the most common cause of sick leave, decreased productivity, and personal financial losses among the different types of compressive neuropathies [3, 4, 6, 13, 14].

Work-related musculoskeletal disorders (WMSDs) among office workers are of interest to researchers because of an increasing incidence [15–18]. Wrist and hand musculoskeletal complaints affected over 15% of occupational groups including office workers, nurses, and others [16]. Office work-related CTS prevalence has been reported to range from nearly 5000 to 7500 per 100,000 individuals [19]. The wrist and hand symptoms are associated with physical factors such as repetitive use of the hands, inappropriate posture during computer work, and lengthy exposure to visual display terminals (VDT) including computer, keyboard, and mouse [3, 6, 8, 20, 21]. Psychosocial variables including high pressure, low decision altitude, and low reward are also estimated to pose potential risks for musculoskeletal complaints [6, 18, 22].

While many studies from different countries have looked at WMSDs in office workers [16, 17, 19], little is known about its current status among the Chinese office workers. According to the National Bureau of Statistics of China, over 3 billion employees have been working in office settings in different sectors nationwide [23]. However, the epidemiology of WMSDs among the vast population remains poorly understood due to insufficient research in this area. The previous few studies mainly focused on neck and back pain, leaving of a gap of knowledge concerning wrist and hand complaints [24–27]. There is an absence of evidence to determine the prevalence of CTS in Chinese office workers and to identify work-related predictors. In light of the research gaps, this study aimed to investigate the prevalence of CTS symptoms involving the wrist and hand regions among officer workers in China and to identify potential risk factors for the development of CTS symptoms in this working population.

## Methods

### Sampling and survey

A cross-sectional survey of a sample of office workers was conducted in the metropolitan city of Guangzhou, China. Based on the official registration statistics of commercial office buildings in Guangzhou, 1030 commercial buildings located in 6 specific administrative districts were identified. Stratified sampling was used, and six district-based subgroups were defined. The buildings in each subgroup were selected through the random number generation method. Thirty office buildings were randomly selected from the 1030 buildings located in different regions of Guangzhou city. The Property Management Offices of the selected 30 buildings were contacted to participate in this survey. The Property Management Office forwarded our research recruitment notice to the respective Human Resource Department that was responsible for circulating the information to their employees. In addition, a specific space was reserved for the research team in each building to conduct the worksite survey during the appointed timeslots that were most appropriate for the majority of workers. Employees at each building were recruited and volunteered as participants. The trained investigator team comprised of 20 research assistants that distributed the questionnaires and supervised the respondents completing the questionnaire after a brief introduction and completion of informed consent. As a bonus for participation in the study, free on-site consultation for musculoskeletal pain as well as a health promotion talk on ergonomics and strategies for occupational hazards prevention was provided after the survey. This study was approved by the Ethics Committee of The Sixth Affiliated Hospital of Sun Yat-sen University before its initiation.

### Questionnaire

A questionnaire was devised based on a validated survey [18], and its content validation was conducted by a multidisciplinary expert panel in piloting prior to the implementation of the survey. The first section included demographic information such as gender, age, height, weight, handedness, educational level, and marital status. The second section was oriented with the specific working conditions of employees, including the department, experience, working hours, and workplace environment and posture. Working departments were classified into departments of Information & Technology, Finance & Accounting, Marketing, Human Resources, Sales, and Other. The experience of the current job was counted by working years. In terms of working hours, weekly working time and daily hours of using the computer and keyboard were collected. The workplace environment was evaluated through a series of questions on use of an adjustable desk and chair, having a break at work, working with vibrating tools, and the overall satisfaction of the workplace. Working posture information was obtained by questions on the positions of wrist and hand, fingers used for mouse control, the distance from eye to the screen, and performing self-adjustment of posture at work. The frequency of working in pain was assessed using the 5-point Likert scale as “never, rarely, sometimes, often, and always”. The third section of the questionnaire focused on the presence of musculoskeletal complaints such as pain and numbness at the wrist and/or hand regions in the past two weeks using a body chart. A numerical scale ranging from 0 to 10 was used to gauge the intensity of the symptoms. The duration and frequency of symptoms, as well as the association with their work were recorded. Occurrence of nocturnal symptoms and aggravating activities were also measured. Questions in the fourth section were about participants’ past medical histories and habits including smoking, physical exercise, and sleep hours. Section five also used a 5-point Likert scale to examine work-related psychosocial factors to explore the level of job demand, decision control, stress at work, and social support.

### Clinical examination

The participants who had reported positive symptoms at the wrist and/or hand region were asked to receive the clinical examinations specifically for CTS. All symptomatic subjects were examined by the same physician who was experienced in performing relevant assessments of CTS. Examination of both hands included the special provocative tests, such as the Tinel nerve percussion test, Phalen’s test, and skin sensation measurements at the wrist and hand region.

Subjects with wrist and/or hand symptoms were identified as having clinically confirmed or clinically uncertain CTS according to their medical history and the findings in the physical examination [1]. Symptomatic participants identified as clinically confirmed CTS met the following criteria: 1) presence of recurring nocturnal and/or activity-related pain, or numbness or tingling involving two or more of the first 4 fingers; 2) positive Tinel percussion test and/or Phalen maneuver. The presence of sensory and/or motor dysfunction related to median nerve was supportive but not necessary for the classification [1, 3]. Those with poorly defined median nerve numbness, or chronic pain as the main complaint were regarded as clinically uncertain CTS [1].

### Boston carpal tunnel syndrome questionnaire

Clinically confirmed CTS participants were asked to complete the Boston Carpal Tunnel Syndrome Questionnaire (BCTQ). The BCTQ is the most widely employed patient-reported outcome measure for CTS [28]. It is comprised of two scales: the Symptom Severity Scale (SSS) and the Functional Status Scale (FSS). The SSS includes 11 questions concerning the nature, frequency, duration of the wrist and hand symptoms during the daytime and nighttime. The FSS contains 8 questions related to the impact of the wrist and/or hand complaints on normal daily activities. Both scales use a five-point rating scale for symptom severity evaluation.

### Statistical analysis

Descriptive statistics of frequencies, percentages, mean, standard deviation, and cross tabulation were used to describe the demographic characteristics. Cross tabulation and Chi-square statistics were used to compare the occurrence rates of symptoms and clinically confirmed CTS. Binary logistic regression was performed to estimate the odds ratio (OR) and 95% confidence interval (95% CI) for the occurrence of self-reported wrist and hand symptoms and clinically confirmed CTS. In addition, comparisons of “symptomatic” versus “asymptomatic” cases and “clinically confirmed CTS” versus “clinically uncertain CTS” were conducted. Univariate logistic regression analysis was carried out for the initial selection of risk factors at the significance level of *p*< 0.10. The variables that showed a significant association with the symptoms in the univariate regression were included in the multivariate logistic regression model. The multivariate model was adjusted for age and gender. All data analyses were conducted using IBM SPSS Statistics for Macintosh, Version 25.0. (Chicago: SPSS Inc.) and the significance level was set at *p*=0.05, except for the univariate analysis, in which a higher *p*-value was set for the purpose of identifying variables in the multivariate model.

## Results

### Demographic information

A total of 1092 questionnaires were distributed to volunteer participants and 969 (610 females and 359 males) completed the valid survey representing a response rate of 89%. The average age of the subjects was less than 30 years (28.5+ 6.39). Approximately 63% of the participants were female and over 90% were right-handed. More than 80% of the participants were diploma level or above. The majority of the respondents were non-smokers and less than half exercised regularly. Between-gender differences were found in lifestyle factors of smoking and exercise (Chi-square test, *p*< 0.001) (Table 1).

**Table 1:** Demographic characteristics of the office workers

Items	Whole group (*n*=969)	Males (*n* = 359)	Females (*n* = 610)
**Age**	28.5 (6.39)	29.2 (5.92)	28.1 (6.63)
**BMI**	20.8 (3.11)	22.2 (3.18)	20.1 (2.79)
**Working experience** (yr)	6.4 (5.99)	6.8 (5.89)	6.1 (1.77)
**Handedness**			
Right-handed	892 (92.1%)	322 (89.7%)	570 (93.4%)
Left-handed	52 (5.4%)	26 (7.3%)	26 (4.3%)
**Education**			
Secondary or below	106 (10.9%)	39 (10.9%)	67 (11.3%)
Diploma level	300 (31.0%)	104 (29.0%)	196 (32.1%)
Bachelor level	418 (43.1%)	172 (47.9%)	246 (40.3%)
Master level or above	120 (12.4%)	39 (10.9%)	81 (13.3%)
**Marital status**			
Single	465 (48.0%)	171 (47.6%)	294 (48.2%)
Married	376 (38.8%)	146 (40.7%)	230 (37.7%)
Divorced	4 (0.4%)	3 (0.8%)	1 (0.2%)
Other	95 (9.8%)	30 (8.4%)	65 (10.7%)
**Smoking**			
Yes	112 (11.6%)	96 (26.7%) *	16 (2.6%) *
No	823 (84.9%)	250 (69.6%)	573 (93.9%)
**Physical exercise**			
Yes	456 (47.1%)	202 (56.3%) *	254 (41.6%) *
No	489 (50.5%)	149 (41.5%)	340 (55.7%)
**Working days per week** (d/w, mean (SD))	5.4 (1.39)	5.4 (0.67)	5.4 (1.68)
**Daily working hours** (hr/d, mean (SD))	8.3 (1.53)	8.4 (1.71)	8.2 (1.41)
**Daily computer use time** (hr/d, mean (SD))	7.9 (2.55)	7.9 (3.03)	7.8 (2.23)
**Daily keyboard use time** (hr/d, mean (SD))	6.2 (2.78)	6.0 (3.09)	6.3 (2.56)
**Sleep hours per day** (hr/d, mean (SD))	7.3 (1.01)	7.2 (0.88)	7.4 (1.07)

Note: Mean values (SD) present for age, BMI, working experience, working days per week, daily working hours, daily computer and keyboard use and sleep hours per day, while handedness, education level, marriage status, smoking and exercise are expressed as counts (% of respective group). * represents *p* < 0.05 and means there were significant differences between men and women workers. *Abbreviations*: *BMI* body mass index, *yr* year, *SD* standard deviation, *d/w* days per week, *hr./d* hours per day

### Occurrence of the wrist and hand complaints

Table 2A shows the occurrence of wrist/hand symptoms. Approximately 60% of the respondents were symptom-free. The prevalence of wrist symptoms including numbness and pain was 22%; 18% of the participants reported hand/finger numbness; and 15% complained of hand/finger pain. No significant differences in wrist and/or hand complaint occurrence were found between female and male office workers. Over a quarter of the participants in the present study reported that the pain limited their ability to work and around 30% had to reduce their leisure activities because of wrist and hand complaints.

**Table 2:** Prevalence of wrist and hand symptoms and clinically confirmed carpal tunnel syndrome

**Wrist/hand symptoms**	**Whole group** (n=969)	**Males** (n = 359)	**Females** (n = 610)
**A Prevalence of wrist and hand symptoms**			
Wrist numbness	217 (22.4%)	90 (25.1%)	127 (20.8%)
Wrist pain	218 (22.5%)	90 (25.1%)	128 (21.0%)
Hand numbness	174 (18.0%)	67 (18.7%)	107 (17.5%)
Hand pain	148 (15.3%)	61 (17.0%)	87 (14.3%)
**B Prevalence of clinically confirmed CTS**			
Asymptomatic	596 (61.5%)	208 (57.9%)	388 (63.6%)
Clinically uncertain CTS	280 (28.9%)	121 (33.7%)	159 (26.1%)
Clinically confirmed CTS	93 (9.6%)	30 (8.4%)	63 (10.3%)

Note: data was expressed as counts (% of respective group)

*Abbreviations*: *CTS* carpal tunnel syndrome

### Prevalence of CTS-related symptoms and signs

The clinically confirmed CTS prevalence is presented in Table 2B. According to the defined criteria for clinically confirmed CTS (1), 9.6% of office workers had CTS, accounting for nearly 25% of the symptomatic workers. The prevalence rates of clinical CTS for male and female workers are 8.4 and 10.3% respectively.

The clinically diagnosed CTS cases were further evaluated with the BCTQ (data not shown). Among the clinically confirmed CTS cases, over half had wrist and hand pain, 60% had wrist and hand numbness, and 32% reported tingling during the day. Thirty percent experienced numbness or tingling discomfort at night and 16% had been woken up at night by numbness or tingling in the past two weeks. Nearly 10% of the cases complained of having difficulty in holding or using small objects. Between 4.3 and 18.5% of those diagnosed with clinical CTS reported difficulty performing daily activities including writing, buttoning, holding things, housekeeping, opening caps, carrying, bathing and dressing; over 15% found it difficult to hold a book, do housekeeping or carry grocery bags.

### Associations between risk factors and positive wrist/hand symptoms

The univariate analyses between symptomatic and asymptomatic cases were used to determine the multivariate regression model (Table 3). After adjusting for age and gender, the multivariate regression analyses showed significant association of wrist/hand symptoms with increased daily computer use time and working without breaks (adjusted ORs: 1.11 (95% CI 1.02 to 1.22) and 1.88 (95% CI 1.12 to 3.14)). In addition, smoking was a significant predictor for positive wrist/hand symptoms (adjusted OR 2.20 (95% CI 1.19 to 4.07)) (Table 4).

**Table 3:** Univariate analyses for wrist/hand symptoms and clinically confirmed CTS

Predictors	Symptomatic (*n*=373) vs. Asymptomatic (*n*=596) OR (95% CI)	Clinically confirmed CTS (*n*=93) vs. Clinically uncertain CTS (*n*=280) OR (95% CI)
**Age**	1.02 (0.99 to 1.04)	1.00 (0.96 to 1.04)
**Gender**		
Male	1	1
Female	1.27 (0.97 to 1.66)	1.70 (1.01 to 2.85)
**BMI**		
	1.07 (1.02 to 1.11)	1.04 (0.96 to 1.14)
**Education**		
Secondary & below	1	1
Diploma	0.37 (0.21 to 0.66)	0.28 (0.06 to 1.27)
Bachelor	0.57 (0.37 to 0.88)	0.58 (0.05 to 7.42)
Master & above	0.89 (0.59 to 1.34)	0.21 (0.04 to 0.99)
**Smoking**		
Yes	1.60 (1.07 to 2.37)	1.22 (0.60 to 2.49)
No	1	1
**Working department**		
Information & Technology	1	1
Finance & Accounting	1.70 (1.18 to 2.44)	0.71 (0.23 to 2.18)
Marketing	0.63 (0.37 to 1.10)	0.66 (0.24 to 1.77)
Human Resources	0.82 (0.49 to 1.36)	0.31 (0.13 to 0.76)
Sales	0.66 (0.40 to 1.07)	0.87 (0.38 to 2.01)
Others	0.65 (0.44 to 0.95)	0.63 (0.33 to 1.20)
**Working experience** (yr)	1.03 (1.00 to 1.06)	0.99 (0.94 to 1.05)
**Daily computer use** (hr)	1.07 (1.01 to 1.12)	0.96 (0.88 to 1.06)
**Daily keyboard use** (hr)	1.05 (1.01 to 1.11)	1.02 (0.93 to 1.12)
**Breaks at work**		
Yes	1	1
No	1.68 (1.15 to 2.44)	1.33 (0.68 to 2.59)
**Adjust posture at work**		
Yes	1	1
No	1.71 (1.24 to 2.36)	1.34 (0.75 to 2.41)
**Working in pain**		
Never	1	1
Rarely	1.61 (1.11 to 2.35)	2.01 (0.55 to 7.24)
Sometimes	1.68 (1.10 to 2.56)	1.82 (0.53 to 6.28)
Often	2.41 (1.47 to 3.94)	3.02 (0.89 to 10.24)
Always	1.81 (0.76 to 4.32)	3.45 (0.94 to 12.73)
**Decision control at work**		
Never	1.87 (1.13 to 3.10)	2.08 (0.46 to 9.36)
Rarely	1.35 (0.84 to 2.18)	1.30 (0.37 to 4.52)
Sometimes	1.32 (0.80 to 2.19)	1.35 (0.39 to 4.62)
Often	0.73 (0.34 to 1.53)	1.53 (0.43 to 5.46)
Always	1	1

*Abbreviations*: *CTS* carpal tunnel syndrome, *BMI* body mass index, *OR* odds ratio, *CI* confidence interval, *yr* year, *hr*. hour

**Table 4:** Multivariate analyses for wrist/hand symptoms and clinically confirmed CTS

Predictors	Symptomatic (n=373) vs. Asymptomatic (n=596) OR (95% CI)	Clinically confirmed CTS (n=93) vs. Clinically uncertain CTS (n=280) OR (95% CI)
**Age**	1.02 (0.97 to 1.07)	1.01 (0.96 to 1.06)
**Gender**		
Male	1	1
Female	1.16 (0.78 to 1.89)	1.51 (0.82 to 2.76)
**BMI**		
	1.02 (0.75 to 1.79)	1.01 (0.92 to 1.12)
**Education**		
Secondary & below	1	1
Diploma	0.46 (0.20 to 1.07)	0.24 (0.05 to 1.14)
Bachelor	0.81 (0.43 to 1.52)	0.48 (0.04 to 6.67)
Master & above	0.61 (0.18 to 2.01)	0.20 (0.05 to 0.97)
**Smoking**		
Yes	2.20 (1.19 to 4.07)	NA
No	1	
**Working department**		
Information & Technology	1	1
Finance & Accounting	1.10 (0.67 to 1.82)	1.51 (0.75 to 3.04)
Marketing	0.48 (0.22 to 1.08)	1.10 (0.36 to 3.42)
Human Resources	0.92 (0.46 to 1.85)	0.88 (0.33 to 2.35)
Sales	0.52 (0.27 to 1.02)	0.57 (0.24 to 1.35)
Others	0.70 (0.42 to 1.16)	1.15 (0.51 to 2.61)
**Working experience** (yr)	1.01 (0.95 to 1.08)	NA
**Daily computer use time** (hr)	1.11 (1.02 to 1.22)	NA
**Daily keyboard use time** (hr)	0.98 (0.90 to 1.06)	NA
**Breaks at work**		
Yes	1	
No	1.88 (1.12 to 3.14)	NA
**Adjust posture at work**		
Yes	1	
No	1.30 (0.88 to 1.92)	NA
**Working in pain**		
Never	1	1
Rarely	1.19 (0.44 to 3.21)	2.53 (0.65 to 9.81)
Sometimes	1.46 (0.56 to 3.82)	2.56 (0.68 to 9.61)
Often	1.82 (0.68 to 4.86)	3.94 (1.06 to 14.64)
Always	1.73 (0.63 to 4.79)	4.73 (1.16 to 19.29)
**Decision control at work**		
Never	1.93 (1.00 to 3.72)	
Rarely	1.55 (0.83 to 2.88)	
Sometimes	1.38 (0.71 to 2.68)	NA
Often	0.76 (0.27 to 2.11)	
Always	1	

Note: The multivariate regression model included those variables with *p <* 0.10 during the respective univariate analyses

*Abbreviations*: *CTS* carpal tunnel syndrome, *BMI* body mass index, *OR* odds ratio, *CI* confidence interval, *NA* not applicable, the factor was not included into the multivariate regression model, *yr* year, *hr*. hour

### Associations between risk factors and clinically confirmed CTS

Through univariate analysis between clinically confirmed CTS and uncertain CTS, statistical association was seen between the female gender and clinical CTS (OR=1.70, 95% CI 1.01 to 2.85). In the multivariate model adjusted by age and gender, “often or always working in pain” was significantly associated with an elevated odd of clinically confirmed CTS (adjusted ORs: 3.94 (95% CI 1.06 to 14.64) and 4.73 (95% CI 1.16 to 19.29)). In addition, higher educational level was associated with lower odds of having clinical CTS (adjusted OR 0.20 (95%CI 0.05 to 0.97)) (Table 4).

## Discussion

This study is the first to report on the relatively high prevalence of wrist/hand pain and clinically confirmed CTS among office workers in China. Those frequently working in pain are more likely to have clinical CTS. Intense use of a computer and working without breaks were found to be associated with increased prevalence of wrist and hand symptoms. Lifestyle may also play a role, as smoking was associated with higher odds of wrist and hand pain after controlling for age and gender.

The occurrence rates of the wrist and hand symptoms were near 22 and 15% respectively. The present study suggests a 10% prevalence of clinically confirmed CTS in Chinese office workers, which is higher than the observations in the western population [12]. The prevalence of CTS in the general population has been reported to be approximately 5% [1, 4–6]. In an earlier cross-sectional study by Andersen et al. [29], 10.9% of office workers had self-reported hand tingling/numbness among computer users, and the clinical interview confirmed median nerve-related CTS symptoms were present in 5% of subjects. Another study reported that self-reported CTS prevalence among office workers in Kuwait was over 18% [30], but it may have been overestimated due to limited convenience sampling. Despite China’s large workforce, the body of literature in the area of musculoskeletal disorders among office workers in China is still limited. The few previous Chinese occupational studies primarily focused on neck and low back pain [25, 26]. Although the prevalence of wrist and hand symptoms is not as high as has been reported for neck or back pain, more than a quarter of respondents in this study stated that the symptoms placed a restriction on their normal work capacity, while 30% had to minimize leisure activities due to wrist/hand disorders. These work-related musculoskeletal symptoms may bring about negative consequences not only in their work productivity but also in quality of life.

While office work-related predictors for CTS have been examined previously [4, 8, 9, 31, 32], a causal relationship between CTS and VDT has not been proven. However, the high prevalence rate of clinically diagnosed CTS and wrist/hand symptoms among Chinese office workers in this study has identified the potential for VDT-related occupational health hazard prevention. With the widespread use of VDT, more attention is needed to potentially reduce computer-using related wrist and hand disorders and CTS in Chinese employees. This study revealed that prolonged daily computer use was a risk factor for wrist and hand pain. Similarly, Cheng et al. found that extended VDT exposure time increased the risk of wrist pain as well as other musculoskeletal discomfort among Chinese office workers [25]. In a meta-analysis, Coenen et al. [33] concluded that increased risks were observed for neck and upper extremity symptoms with intensive exposure to VDT-related tasks. Increased carpal tunnel pressure was also noted with computer mouse use which may be a mechanism in developing CTS among computer workers [21, 31]. Considering the lack of comparison between VDT-using and non-VDT-using office workers, future well-designed studies on screen work-related factors for CTS are warranted.

The odds of employees being diagnosed with CTS who often or always worked while in pain were 3.94 to 4.73 times greater than those did not experience any pain at work. A lack of breaks at work was also associated with increased odds of positive wrist and hand complaints. High levels of repetitive and forceful exertion are associated with an elevated hazard of CTS [4, 6, 32]. Working for prolonged periods without rest intervals may lead to physical overload due to sustained static postures or repetitive wrist and hand exertions, resulting in mental distress [34].

It can be assumed that those who need to work with pain frequently may experience more physical and mental stress at work without adequate rest, which may in turn aggravate the symptoms and increase the risk of CTS. The competitive economic environment in modern industries makes overtime work culture such as a “996” schedule (working from 9:00 am to 9:00 pm, 6 days a week) a routine for many corporations in China. Employees under such circumstances may not be able or willing to take absence from work even with painful symptoms. Even though the exact sickness absence rate of participants involved in this study was not evaluated, some human resource managers noted that only a small number of their staff had asked for sick leave in the past year. Previous studies have reported that a large majority of Chinese workers did not request to go on sick leave after reporting musculoskeletal pain [35, 36].

Gender and body mass index (BMI) are the commonly reported individual factors contributing to CTS [1, 4, 11]. Women seem more likely to experience CTS, which may be related to hormonal and circulatory factors [6]. The annual incidence for females in Sweden was 428 cases per 100,000, compared with 182 per 100,000 for males [37]. The odds for women to develop either CTS symptoms or CTS confirmed by nerve conduction studies were almost 2 times that for men [9]. Obesity has also been identified as a risk factor for CTS [6, 38]. Elevated BMI has been associated with the severity of CTS symptoms [39]. Although an association was seen between female gender and clinical CTS in the univariate analysis of this study, gender was not a significant risk factor for CTS in the multivariate model. Likewise, BMI was associated with the presence of wrist and hand symptoms in the univariate regression; however, it was not significant in the multivariate model adjusted for age and gender. Several reasons may explain these discrepancies. First, the lack of neurophysiological examination of the median nerve in the present study may result in some false positive CTS cases. Second, the width of the confidence intervals suggest that the sample size may have been not large enough to detect significant associations.

Other aspects affecting wrist and hand pain or CTS in this study included smoking and educational background. Smokers in this study were more likely to experience wrist and hand symptoms than non-smokers. Similarly, other researchers found significant associations between smoking and musculoskeletal disorders or CTS [10, 40]. The risk for CTS in young (< 31 years old) females reporting heavy smoking (> 10 pack years) was about 2 times greater than non-smokers [10]. Smoking has been shown to adversely affect the blood flow of the sciatic nerve in an animal model, resulting in nerve degeneration and fibrosis [41]. Higher educational level (Masters and above) appeared to be a protective factor for CTS in this study. One possible assumption is that higher-educated personnel may be exposed to less physically demanding or repetitive work that may increase risk for CTS.

Both limitations and strengths exist in the study. First, a nerve conduction test (the gold standard for CTS diagnosis) was not included in the present study, which was one of the major limitations of the study. Researchers note that CTS-related symptom severity is not correlated with the incidence of a confirmed CTS diagnosis [42, 43]; and, among subjects complaining CTS symptoms, those not diagnosed through nerve conduction velocity report higher anxiety scores [14]. Another limitation is that work-related factors were not evaluated using objective measures in this study. Thus, the self-reported exposure variables may be subject to misclassification bias. Future studies employing objective quantitative measures of exposure should be performed.

This study was also limited by design bias. The cross-sectional study design limited the exploring of longitudinal effect of predictors on CTS. With a block-randomized design, the subjects responding to this survey were limited to those located in the buildings chosen throughout the city, which might lead to selection bias. An on-site occupational study could as well be biased by the well-known “healthy worker effect” [44], although the sickness absence rate based on a register of all employees seems relatively low in Chinese employees [35, 36]. Attempts were made to attract as many workers as possible by negotiating suitable timeslots for the survey and by providing additional benefits including free consultation and occupational health talks.

One of the strengths of the study was the relatively large sample size of 969 participants that was carried out in a Chinese metropolitan city. This improves the generalizability of the results to office workers in major cities of China. The valid response rate of the survey was satisfactory (89%), which minimizes the response bias. Furthermore, the findings of the present study were based on a relatively young age group, which may shed light on the potential of early-aged CTS development. The rather high prevalence of clinically confirmed CTS and wrist/hand complaints in this study should urge company managers, administrators, and office workers to raise awareness of the industrial risks of CTS and wrist and hand symptoms. Specific prophylactic programs for occupational health are encouraged to be researched and implemented to minimize severe health hazards in this population.

## Conclusions

In conclusion, relatively high prevalence rates of work-related wrist and hand complaints and clinically confirmed CTS were seen among young office workers in China. Those frequently working with pain show higher odds for having CTS, and prolonged computer use and working without breaks are risk factors for wrist and hand symptoms. Smokers were more likely to report wrist and hand complaints, while higher education level seemed to be a protective factor for CTS. Future preventative and interventional studies in the workplace need to consider these risk factors.

## Data Availability

The datasets used and/or analyzed for the current study are available from the corresponding author on reasonable request for academic purpose.

## Abbreviations

CTS: Carpal tunnel syndrome
WMSDs: Work-related musculoskeletal disorders
BMI: Body mass index
VDT: Visual display terminals
BCTQ: Boston carpal tunnel syndrome questionnaire
SSS: Symptom severity scale
FSS: Functional status scale
OR: Odds ratio
CI: Confidence interval
“996”: working from 9:00 am to 9:00 pm, 6 days a week
